# Predictors of attendance at an obesity clinic and subsequent weight change

**DOI:** 10.1186/1472-6963-14-78

**Published:** 2014-02-20

**Authors:** Emma Brook, Lauren Cohen, Paul Hakendorf, Gary Wittert, Campbell Thompson

**Affiliations:** 1 University of Adelaide, Adelaide, South Australia, Australia; 2 Clinical Epidemiology, Flinders Medical Centre and Flinders University, Adelaide, South Australia, Australia; 3 Department of Medicine, Royal Adelaide Hospital and University of Adelaide, Adelaide, South Australia, Australia

**Keywords:** Body mass index, Attrition, Depression, Rural residence, Obstructive sleep apnoea, Evaluation

## Abstract

**Background:**

There is conflicting evidence regarding characteristics of patients most likely to have poor outcomes after referral to a multidisciplinary weight loss clinic. The aim of this study was to identify patient characteristics associated with poor attendance and poor weight outcomes at a weight management clinic based in an Australian tertiary hospital.

**Methods:**

Patient characteristics including age, sex, referral source, postcode of residence, weight, body mass index (BMI) and the presence of specific comorbidities were recorded. Outcome measures included questionnaire return following referral (a requirement prior to a first appointment being scheduled), percentage of appointments attended and rate of weight change (kg/month). Continuous variables were expressed as mean ± standard deviation and compared using a *t*-test. Categorical data were presented as proportions and a chi-squared test was used to test significance. Statistical significance was set as p < 0.05.

**Results:**

Of 502 patients referred to the Comprehensive Metabolic Care Centre (CMCC), 231 (46%) did not return their questionnaire. Patients referred by their GP, compared to those with only internal hospital referrals, were more likely to return their questionnaire (86.0% cf. 77.9%; p = 0.02) as were those who had their BMI recorded in their referral letter (58% cf 45% p = 0.011). 28.1% of patients attended half or less of their scheduled appointments at the CMCC but none of the parameters analysed was associated with attendance. Weight loss was associated with residence in a rural location (p = 0.016) and hypercholesterolaemia (p = 0.03) and weight gain was associated with obstructive sleep apnoea (p = 0.04).

**Conclusions:**

A large proportion of the patients referred to a weight management clinic never had an appointment scheduled. Clinicians should not anticipate greater compliance in one patient demographic than another; all groups need focus, particularly at the referral stage, and likely poor compliance must be anticipated and better managed.

## Background

The high prevalence of obesity and associated complications presents both a significant service and direct cost burden to the Australian Health Care system [1,2]. There are also substantial indirect socioeconomic costs associated with obesity, including reduced workforce participation and loss of productivity [3]. The recent trend for a disproportionate increase in severe obesity, often with multiple complications, has prompted a renewed focus on services in tertiary care settings, where in accordance with available evidence, multidisciplinary clinics have been established [4-6]. High attrition rates [4,7-9] and poor weight loss outcomes have been well documented for these clinics [4,10] although the patients most likely to develop these poor outcomes have not been clearly and consistently defined [4,7,10]. For example, attrition in some studies has been linked to psychological ill health and lower gross family income [8,11], whereas other studies have shown higher attrition rates in patients in full-time employment, and improved attendance by patients with a history of depression [7,10]. Early identification of patients who fail to attend or continue to gain weight might allow for the institution of an alternate process of care that optimizes all patients’ outcomes. We therefore aimed to determine patient characteristics associated with poor attendance and poor weight outcomes at a weight management clinic based at a tertiary hospital.

## Methods

This was a retrospective observational cohort study that involved all patients presenting to the Comprehensive Metabolic Care Centre (CMCC) at the Royal Adelaide Hospital (RAH) from October 2010, to November 2012, and all referrals to the clinic from December 2011, to November 2012. Approval for this study was obtained from the RAH Research Ethics Committee (approval no. 120101). The CMCC is a multi-disciplinary clinic staffed by physicians, bariatric surgeons, dieticians and exercise physiologists. It operates for half a day each week, taking referrals from South Australian General Practitioners (GPs) and clinicians working in public hospital outpatient departments. Following referral to the clinic, each patient is sent informative materials about the services offered by the CMCC and a nine page questionnaire to complete. In order for an appointment to be scheduled, the questionnaire needs to be returned and the patient must call and make the appointment personally. Patients are reviewed by a physician at their first appointment, which may be up to nine months from referral, with subsequent appointments with a dietician and exercise physiologist. A small proportion of patients are referred to a surgeon for consideration of bariatric surgery.

### Measures

Demographic information and anthropometric measurements were obtained from the referral letter and case notes, including age, sex, referral source, postcode of residence, weight and body mass index (BMI). For each patient, postcode of residence was used to determine rural or urban status and their index of relative socioeconomic disadvantage (IRSD) as defined by the Australian Bureau of Statistics [12]. The presence of the following comorbidities was determined based on information in the referral letter and clinic notes: hypertension, hypercholesterolemia, hypothyroidism, obstructive sleep apnea (OSA), ischaemic heart disease, depression and diabetes mellitus.

For each patient referred we recorded whether a questionnaire was returned and whether the ensuing appointment was attended. In addition, attendances at follow-up appointments were compared with appointments scheduled. Changes in patients’ weights were expressed as the rate of weight change (kg/month) and assessed relative to the weight recorded at their first clinic visit. Due to the variable number of appointments scheduled for each patient, attendance and rate of weight change were calculated based on data for the first appointment and the last appointment recorded for that patient.

### Statistics

Statistical analysis was performed using STATA 12.0 (StataCorp, Texas, USA). Continuous variables were expressed as mean ± standard deviation and compared using a *t*-test. Categorical data were presented as proportions and a chi-squared test was used to test significance. Statistical significance was set as p < 0.05.

## Results

### Patient characteristics and demographics

#### *Clinic referrals*

A total of 502 patients, 69.3% female, were referred between December 2011 and November 2012. The average age of males was 46.0 years (s.d = 12.7) and of females 43.6 years (s.d = 14.2), with the range of ages varying from 17 years to 82 years old. 37.7% of males and 31.0% of females were from rural locations. The mean IRSD decile was 3.47 (s.d = 2.52) for males and 3.53 (s.d = 2.56) for females. The majority (82.3%) of referrals to the clinic were made by GPs. Only 60.4% of referral letters (303 letters) contained a weight and 69.7% (350 letters) contained a BMI. From the available data, males were heavier than females (mean weight and BMI for males was 158.1 kg (s.d = 25.9) and 50.3 kg/m^2^ (s.d = 8.2) respectively; for females mean weight was 133.3 kg (s.d = 27.1) and mean BMI was 47.9 kg/m^2^ (s.d = 9.1)).

#### *Scheduled appointments and attendance*

Of the 502 patients referred to the CMCC, 231 (46%) did not return their questionnaire and therefore no appointment was made. Between October 2010 and November 2012, appointments were scheduled for 392 patients whose characteristics were not different from the referred cohort. Females comprised 70.4% of this smaller group. The average ages for males and females were 49.6 years (s.d = 11.7) and 45.6 years (s.d = 13.1) respectively (age range from 20 years to 76 years), and 35.3% of males and 29.7% of females were from rural locations. The mean IRSD decile was 3.91 (s.d = 2.59) for males and 3.59 (s.d = 2.61) for females. The mean weight and BMI as recorded at the first clinic visit for males was 160.7 kg (s.d = 32.2) and 51.4 kg/m^2^ (s.d = 9.5) respectively. For females, the mean weight was 129.9 kg (s.d = 25.6) and mean BMI was 48.5 kg/m^2^ (s.d = 8.7).

### Predictors of returning the questionnaire

Patients referred by their GP, compared to those with only internal hospital referrals from other medical specialists, were more likely to return their questionnaire (86.0% cf. 77.9%; p = 0.02). Those who had a BMI recorded in their referral letter were more likely to return their questionnaire (58% c.f 45% p = 0.011). The prevalence of diabetes in those returning their questionnaire was 33.6% as compared to 25.5% in those not returning their questionnaire (p = 0.05). There were no significant differences between the two groups for any other measured parameter (Table 1).

**Table 1:** Predictors of those who do not return their questionnaire after referral

	**Whole population**	**Questionnaire returned**	**Questionnaire not returned**	**P Value**
**Number of patients**	502	271	231	-
**Age**				
Mean (SD) age (yrs)	44.3 (13.8)	45.2 (13.7)	43.3 (13.8)	0.14
< 35 years, % (n)	27.49 (138)	26.2 (71)	29.0 (67)	0.71
35-44 years, % (n)	24.70 (124)	22.88 (62)	26.84 (62)	0.61
45-54 years, % (n)	23.31 (117)	23.62 (64)	22.94 (53)	0.08
≥55 years, % (n)	24.5 (123)	27.31 (74)	21.21 (49)	0.08
**Female gender, % (n)**	69.3 (348)	70.8 (192)	67.5 (156)	0.42
**Rural postcode, % (n)**	33.1 (166)	35.4 (96)	30.3 (70)	0.22
**Mean IRSD Decile (SD)**	3.51 (2.54)	3.56 (2.51)	3.46 (2.59)	0.67
**GP referral, % (n)**	82.3 (413)	86.0 (233)	77.9 (180)	0.0185
**Mean weight at referral (kg) (SD)**	141.1 (29.1)	140.7 (29.2)	141.7 (29.0)	0.92
**(**Weights recorded for 303 patients)				
**Mean BMI at referral (SD)**	48.6 (0.47)	48.3 (0.63)	49.0 (0.72)	0.40
**(**BMI recorded for 350 patients)				
**Comorbidities, % (n)**				
Hypertension	26.1 (131)	28.4 (77)	23.4 (54)	0.20
High cholesterol	14.1 (71)	16.2 (44)	11.7 (27)	0.15
Hypothyroidism	10.2 (51)	9.2 (25)	11.3 (26)	0.45
OSA	16.5 (83)	18.8 (51)	13.9 (32)	0.14
IHD	3.8 (19)	2.6 (7)	5.2 (12)	0.13
Depression	25.7 (129)	27.3 (74)	23.8 (55)	0.37
DM	29.9 (150)	33.6 (91)	25.5 (59)	0.0499
**Mean total no. of comorbidities (SD)**	1.26 (1.25)	1.36 (1.23)	1.15 (1.27)	0.0557

*SD* standard deviation, *IRSD* index of relative socioeconomic disadvantage, *OSA* obstructive sleep apnea, *IHD* ischaemic heart disease, *DM* diabetes mellitus.

### Predictors of attendance at clinic

28.1% of patients attended half or less than half of their scheduled appointments at the CMCC. In addition, of all those referred to the clinic who had a first appointment scheduled, 17.1% did not attend. None of the parameters analyzed was associated with attendance (Table 2).

**Table 2:** Predictors of attendance rate for all appointments for each patient

	**Whole population**	**Attended >50% of appointments**	**Attended ≤50% of appointments**	**P Value**
**Number of patients**	392	289	103	-
**Age**				
Mean (SD) age (yrs)	46.5 (12.8)	46.4 (12.8)	46.9 (13.0)	0.74
< 35 years, % (n)	19.64 (77)	20.42 (59)	17.48 (18)	0.22
35-44 years, % (n)	22.7 (89)	22.84 (66)	22.33 (23)	0.96
45-54 years, % (n)	29.08 (114)	27.68 (80)	33.01 (34)	0.57
≥55 years, % (n)	28.57 (112)	29.07 (84)	27.18 (28)	0.85
**Female gender, % (n)**	70.4 (276)	68.9 (199)	67.0 (77)	0.26
**Rural postcode, % (n)**	31.4 (123)	33.2 (96)	26.2 (27)	0.19
**Mean IRSD Decile (SD)**	3.69 (2.60)	3.64 (2.55)	3.82 (2.75)	0.55
**Mean weight at 1st appointment (kg) (SD)**	139.0 (31.0)	139.7 (30.8)	137.0 (31.6)	0.41
**Mean BMI at 1st appointment (SD)**	49.4 (9.0)	49.4 (8.9)	49.2 (9.3)	0.86
**Comorbidities, % (n)**				
Hypertension	49.6 (194)	49.0 (141)	51.5 (53)	0.66
High cholesterol	30.4 (119)	31.3 (90)	28.2 (29)	0.56
Hypothyroidism	12.3 (48)	11.5 (33)	14.6 (15)	0.41
OSA	33.4 (131)	34.0 (98)	32.0 (33)	0.71
IHD	7.9 (31)	8.7 (25)	5.8 (6)	0.36
Depression	41.4 (162)	41.7 (120)	40.8 (42)	0.88
DM	29.4 (115)	30.6 (88)	26.2 (27)	0.41
**Mean total no. of comorbidities (SD)**	2.05 (1.53)	2.07 (1.56)	1.99 (1.45)	0.67

*SD* standard deviation, *IRSD* index of relative socioeconomic disadvantage, *OSA* obstructive sleep apnea, *IHD* ischaemic heart disease, *DM* diabetes mellitus.

### Predictors of rate of weight change

Data regarding rate of weight change between first appointment and last recorded appointment were available for 224 patients. Of these, 74 gained weight (mean = 0.56 kg/month; s.d = 0.60 kg/month) between the first and last recorded appointment, 123 lost weight (mean = 0.78 kg/month; s.d = 1.0 kg/month) and 27 remained weight stable (lost or gained less than 0.1 kg/month). When weight loss was analyzed as a continuous variable, weight loss was associated with residence in a rural location (p = 0.016) but age, sex, IRSD decile and other comorbidities were not associated with change in weight (data not shown). When patients were categorized into two groups on the basis of whether they lost weight (n = 123) or did not (n = 101), the place of residence was not a significant predictor of presence or absence of weight loss but those with hypercholesterolemia were more likely to lose weight and those with obstructive sleep apnea less likely to lose weight (Table 3).

**Table 3:** Predictors of weight change (kg/month) from 1st appointment to last appointment

	**Whole population**	**Lost weight**	**Did not lose weight**	**P Value**
**Number of Patients, % (n)**	100 (224)	54.9 (123)	45.1 (101)	-
**Weight change (kg/month) (SD)**	-0.25 (1.02)	-0.78 (1.0)	0.41 (0.57)	-
**Age**				
Mean (SD) age (yrs)	47.2 (12.6)	47.1 (12.6)	47.4 (12.7)	0.86
< 35 years, % (n)	18.3 (41)	17.89 (22)	18.81 (19)	0.94
35-44 years, % (n)	20.09 (45)	22.76 (28)	16.83 (17)	0.63
45-54 years, % (n)	30.8 (69)	28.46 (35)	33.66 (34)	0.64
≥55 years, % (n)	30.8 (69)	30.89 (38)	30.69 (31)	0.99
**Mean IRSD Decile (SD)**	3.77 (2.59)	3.52 (2.41)	4.08 (2.78)	0.11
**Mean total no. of comorbidities (SD)**	2.01 (1.59)	1.98 (1.65)	2.05 (1.52)	0.73
**Female gender, %**	67.4	67.5	67.3	0.98
**Rural postcode, %**	29.0	31.7	25.7	0.33
**Comorbidities, % (n)**				
Hypertension	48.2 (108)	50.4 (62)	45.5 (46)	0.47
High cholesterol	28.1 (63)	34.1 (42)	20.8 (21)	0.03
Hypothyroidism	12.5 (28)	9.8 (12)	15.8 (16)	0.17
OSA	35.3 (79)	29.2 (36)	42.6 (43)	0.04
IHD	8.0 (18)	9.8 (12)	5.9 (6)	0.30
Depression	39.7 (89)	35.8 (44)	44.6 (45)	0.18
DM	29.0 (65)	28.5 (35)	29.7 (30)	0.84

*SD* standard deviation, *IRSD* index of relative socioeconomic disadvantage, *OSA* obstructive sleep apnea, *IHD* ischaemic heart disease, *DM* diabetes mellitus.

To determine whether failure to lose weight was related to subsequent non-attendance, weight change between appointments 1 and 2 was compared to attendance at appointment 3. 173 patients had a third appointment scheduled. Of these, 126 patients (72.8%) attended their scheduled appointment. No statistically significant association was found when comparing weight change with attendance at the third appointment (p values >0.05).

## Discussion

This study has identified a relationship between referral source and questionnaire return, with patients being referred by their GP having a higher rate of questionnaire return. The amount of information in that referral might also be indicative of patient engagement because the recording of BMI in the patient referral letter was also associated with increased likelihood of that patient returning their questionnaire. Although there are very few studies that examine the relationship between referral source and engagement, at least one study conducted in South Carolina has shown that physician-referred patients, compared to those who were self-referred, were more likely to attend the initial introductory consultation for weight management [13]. The management of chronic diseases is dependent on well-coordinated and comprehensive care [14] and our data highlight the importance of general practitioner involvement in the processes of referral and ongoing care.

Almost half of the patients referred to the clinic failed to return the questionnaire and therefore did not have an appointment scheduled. This is consistent with a prior study showing that 33% of patients referred (by either a general practitioner or hospital healthcare staff) to a weight management clinic did not book an appointment [4]. We are unable to establish any demographic or patient factors that predict people who do not return the questionnaires. Binks and O’Neil [13] showed that although physician-referred patients were more likely to attend the initial complimentary consultation, they were less likely to enroll in a specific treatment program than those patients who were self-referred. This was thought to relate to reduced motivation and financial considerations in the physician-referred group [13]. Whilst there are few other studies in the literature examining this alarming attrition rate in the context of weight management clinics, studies of attendance following referral to other medical clinics have implicated the role of full time employment and job commitments, lack of understanding about the need for the appointment and long waiting time before a first appointment is scheduled, as barriers to making an appointment following referral [15]. In our patients attending the CMCC, it is possible that a discrepancy exists between what the clinic offers and what patients expect. As a result, the informative materials explaining the role of the clinic to patients do not encourage them to make an initial appointment. To explore this issue and the motivation of non-attendees, further research would be beneficial seeking feedback from those not returning their questionnaire.

The rate of weight loss of patients attending our clinic was no greater than the rate of weight loss reported by others [4] suggesting our referral process did not generate a significantly more motivated or compliant outpatient population to compensate for the high attrition rate. Rural location of residence was associated with a greater rate of weight loss while attending the clinic. Specific reasons for this have not been established in this study. These patients must travel farther to appointments (over 40 km) and this may reflect a greater commitment by these patients to any prescribed weight loss strategy. Previous studies have shown that those who reside a greater distance from the hospital are significantly less likely to attend clinic appointments [11,16]. Our study, comparing rural and urban patients, has not supported such a trend presumably because the distances travelled from rural South Australia to Adelaide are significantly greater than the average 14 km reported in this previous work.

Previous studies of obese pediatric populations have found low socioeconomic status to be a potential barrier to attendance [11,17]. In our study, IRSD did not associate with attendance. Our finding that age, gender and baseline anthropometric measurements were not significant predictors of treatment attendance, is consistent with some previous studies [7,17] but contrasts with a study by Honas et al. [9] which found younger age to be associated with drop-out from a weight management clinic. Inelmen et al. [7] found that patients with fewer obesity-related diseases were more likely to drop out of a treatment program. The number of co-morbidities did not associate with clinic attendance in our study. In addition, higher BMI has been linked to non-attendance in previous studies examining attendance rates at a range of chronic disease clinics [18,19]. However, our study did not find an association between BMI and attendance – but the BMIs of patients in our study exceeded those of most patients referred to other chronic disease management clinics. Other potential reasons for discrepancies between our data and these studies may relate to differences in the definition of attendance. Others have defined attendance according to completion or non-completion of a specific treatment program, whereas we have defined attendance as the percentage of appointments attended.

Our study has identified a high prevalence of depression (41.4%) in patients attending the clinic, a similar rate to that shown in patients with advanced cancer [20]. In comparison, national data reported by the Australian Institute of Health and Welfare in 2010 indicated that in those aged 16–85 years old, only 6% suffered from an affective (mood) disorder [1]. The association and reciprocal link between depression and obesity has been well documented, with one meta-analysis showing that obese patients had a 55% increased risk of developing depression and depressed patients had a 58% increased risk of becoming obese [21]. The role of depression in obesity treatment attendance and performance is uncertain; some studies show poorer attendance amongst depressed patients [8,11] and others show improved attendance in those with a history of depression [7,10]. It has been suggested that depression motivates help-seeking for weight loss in the belief that it will improve self-esteem and mood [7,10]. In one small study, the presence of depression appeared to increase compliance with treatment but the probability of weight loss was lower in these patients [10]. Depression was unrelated to clinic attendance and weight change in our larger study. We used a looser but perhaps more clinically relevant definition of depression.

There was no effect of gender on return of questionnaires, attendance or treatment outcomes, although, as with other studies, females are over-represented in the sample of patients being referred to weight management services [4,13]. In 2007–08, data from Australian adults indicated that 42.2% of males and 31.1% of females were considered overweight and 25.4% of males and 23.7% of females were considered obese [1]. The lower rate of referral of males to weight management clinics requires attention. Previous studies have suggested that there is reluctance amongst males to seek any medical advice and this may be related to biological and psychological factors as well as social traditions [22,23].

Our results have provided information regarding the prevalence of comorbidities in an obese population, highlighting the poorer health of this group of patients. Compared to national data detailing the prevalence of comorbidities in Australians aged ≥25 years old [1], patients attending our weight management clinic had a much higher prevalence of hypertension, diabetes, depression and ischaemic heart disease. The high number of comorbidities occurring in obese patients confirms the importance of a multi-disciplinary approach to obesity management. The presence of sleep apnea impairing weight loss has been recognized previously [24] and our observation that hypercholesterolemia improves the chances of the patient losing weight merits further study. In addition, given the high prevalence of depression reported in our patient population, a psychologist should be included on any multi-disciplinary team addressing weight management.

There are several limitations to this study that should be noted. Firstly, barriers to appointment attendance and poor weight loss outcomes focussed on specific patient demographics and patient comorbidities. However, other important barriers (eg. Self-efficacy, family stress, adherence to dietary and exercise recommendations, travel distance to clinic) were unable to be recorded and included for each patient, preventing further evaluation of predictors of attendance and weight loss. Secondly, there was not a consistent number of visits scheduled for each patient, so comparisons between patient attendance had to based on percantage of appointments attended. Thirdly, the majority of patients included in this study were severely obese (mean BMI >45 kg/m^2^), suggesting that these study findings are most applicable to obese rather than overweight patients.

## Conclusions

In conclusion, our study has identified that a large proportion of the patients referred to a weight management clinic never have an appointment scheduled and a smaller, significant but also unpredictable proportion who do attend once, have poor ongoing attendance and poor weight loss outcomes. Although GP-referred patients were more likely to return the questionnaire, the high number overall of patients failing to return their questionnaire to the clinic indicates the referral process is critical for the engagement of patients so that they make initial contact with the clinic. Our findings are important because clinicians must not expect that a patient who has been referred to our weight management clinic will attend that clinic. In fact, referred patients who attend the clinic on at least one occasion are in the minority. Clinicians should not anticipate greater compliance in one patient demographic than another nor focus their attentions upon certain groups of obese patients; all groups need focus and likely poor compliance must be anticipated and better managed. Our study has also identified a need to refer more males to weight management services and has reiterated the importance of psychologist and general practitioner involvement in addressing weight issues.

## Competing interests

No competing interests were declared.

## Authors’ contributions

LC contributed to data collection, its analysis and revision of the manuscript. PH contributed to data analysis, interpretation and revision of the manuscript. CT, GW and EB contributed to the study design, data analysis and interpretation and the writing and revision of the manuscript. All authors read and approved the final manuscript.

## Pre-publication history

The pre-publication history for this paper can be accessed here:

http://www.biomedcentral.com/1472-6963/14/78/prepub
